# Piracema: a *Phishing* snapshot database for building dataset features

**DOI:** 10.1038/s41598-022-19442-8

**Published:** 2022-09-07

**Authors:** Julio Cesar Gomes de Barros, Carlo Marcelo Revoredo da Silva, Lucas Candeia Teixeira, Bruno José Torres Fernandes, Joao Fausto Lorenzato de Oliveira, Eduardo Luzeiro Feitosa, Wellington Pinheiro dos Santos, Henrique Ferraz Arcoverde, Vinicius Cardoso Garcia

**Affiliations:** 1 Escola Politécnica de Pernambuco (POLI), Universidade de Pernambuco (UPE), Recife, PE 50720-001 Brazil; 2 Instituto de Computação (IComp), Universidade Federal do Amazonas (UFAM), Manaus, AM 69080-900 Brazil; 3 Departamento de Engenharia Biomédica (DEBM), Universidade Federal de Pernambuco (UFPE), Recife, PE 50740-560 Brazil; 4 Centro de Informática (CIn), Universidade Federal de Pernambuco (UFPE), Recife, PE 50740-560 Brazil

**Keywords:** Computer science, Software

## Abstract

Phishing is an attack characterized by attempted fraud against users. The attacker develops a malicious page that is a trusted environment, inducing its victims to submit sensitive data. There are several platforms, such as PhishTank and OpenPhish, that maintain databases on malicious pages to support anti-phishing solutions, such as, for example, block lists and machine learning. A problem with this scenario is that many of these databases are disorganized, inconsistent, and have some limitations regarding integrity and balance. In addition, because phishing is so volatile, considerable effort is put into preserving temporal information from each malicious page. To contribute, this article built a phishing database with consistent and balanced data, temporal information, and a significant number of occurrences, totaling 942,471 records over the 5 years between 2016 and 2021. Of these records, 135,542 preserve the page’s source code, 258,416 have the attack target brand detected, 70,597 have the hosting service identified, and 15,008 have the shortener service discovered. Additionally, 123,285 records store WHOIS information of the domain registered in 2021. The data is available on the website https://piracema.io/repository.

## Background and summary

Phishing is a type of social engineering attack where the attacker develops a fake page that presents itself as a trusted environment, inducing its victims to submit sensitive data, such as, for example, access credentials to a certain genuine service^1^. The word “phishing” first emerged in the year 1996, when criminals stole passwords from American Online (AOL) users^2^. When analyzing the timeline of phishing attacks, one can see an evolution from generic fraud attempts without defined targets to exploitation based on trends, facts, and opportunities. In other words, attackers have realized that the more valuable the target, the more resources (mostly money) they raise in an attack campaign^3^.

According to Kaspersky report^4^, in 2019, attacks of this type caused monetary losses close to 1.7 billion dollars. In 2020, the number of phishing attacks doubled during the first month of the quarantine^5^, reflecting the need to digitize companies and the mass migration of small businesses to e-commerce at the time. In 2021, the number of attacks continued to increase, with a 50% increase in attacks over the previous year^6^.

In the fight against phishing attacks, numerous solutions have been proposed^3,7,8^. Currently, those that adopt machine learning techniques (Machine Learning) have grown in number and importance^9–11^. However, a noticeable problem in machine learning solutions in many areas is a need (dependence) on datasets to train and test. For example, Tang et al.^12^ employ an old (built in 2012) and unbalanced dataset, called Drebin, to detect security vulnerabilities in Android applications. Similarly, Qi et al.^13^ proposed a novel privacy-aware data fusion and prediction approach for the smart city industrial environment tested in a dataset built in 2012. Already Ma et al.^14^ create a dataset to identify cybersecurity entities in unstructured text. However, how collected the data and why were chosen are not described. These aspects difficult the reproducibility in other works.

According to Allix et al.^15^, machine learning models learn from the input data, and the impact of their performance (i.e., predictive capacity) is related to the datasets (set data) used for training. Therefore, an “adequated” dataset must-have features such as complete data, actuality, and diversity. Li et al.^16^ state that reduced datasets (manually verified) are generally used to evaluate anti-phishing mechanisms. This is due to the various inconsistencies that can appear in large repositories, such as PhishTank, which does not have a cleaning mechanism to remove invalid and offline URLs, resulting in an incorrect database.

The problem is how to create this “adequated” dataset? A dataset is defined by an expert analyst who designs more specific structured information. These informations are from a repository values the volume of evidence, that is, with a more general purpose, thus enabling the building of distinct datasets from the same repository.

Analyzing many of the phishing datasets available (someones discussed in related work section), it notes that almost then obtained their data from phishing platforms like PhishTank, OpenPhish, and PhishStats. Although academic and commercially recognized, these platforms present problems such as, for example, data disorganization (lack of format), data inconsistency, and lacking information (null or absent values). For these reasons, before building a dataset, it is necessary to pre-treat the database information to ensure that the analysis results are consistent and unbiased. To address this problem, this article presents a public phishing database, organized and consistent, to help studies that need to build a dataset. The base was used previously in the study by Silva et al.^17,18^, analyzing static and dynamic aspects of phishing, which justifies its relevance for data reuse. By storing information such as WHOIS and page content, the base includes details regarding temporal aspects, i.e., phishing behaviors that can be loosed over time due to their volatile nature, which is why this base receives the term snapshot. The database records phishing incidents from 2016 to 2021, totaling 942,471 records.

## Related works

Works like Roy et al.^19^, Shantanu et al.^20^, Al-Ahmadi et al.^21^, Alkawaz et al.^22^, Al-Ahmadi^23^, Orunsolu et al.^24^, and others, propose new methods of detecting phishing pages using machine learning methods (Random Forest—RF, Recurrent Neural Networks—RNN, Support Vector Machine—SVM, K-Nearest Neighbours—KNN, and Multilayer Perceptron—MLP). Although they use different classifiers, these works tipically train your models in your own datasets created from PhishTank, OpenPhish, and other platforms.

The problem with these works is the lack of information about the process and steps of building these datasets. The same can be said when comparisons are made between these datasets and pages of the repositories. In addition to the lack of information regarding the construction, which makes it impossible to reproduce the datasets, the non-disclosure of the instances (samples) collected and used also makes a more realistic comparison impossible. For example, is fair compar a dataset built with phishing pages released in 2022 with existing pages in a repository released in 2012? Are there the same characteristics in both of the data?

Based on the above, it is possible to observe that these studies needed to create or seek a knowledge base to evaluate their proposals. The purpose of Piracema is to offer a repository with a volume of information capable of showing patterns in phishing attacks. We believe that many researchers, who aim to mitigate phishing attacks, will have well-structured, information-rich, and integrated data to build their datasets for their intelligent solutions. Given this, we believe that our proposal is an excellent contribution to the Open Source Intelligence (OSINT) scenario for the academic community that wants to combat Web fraud.

## Methods

**Figure 1:**
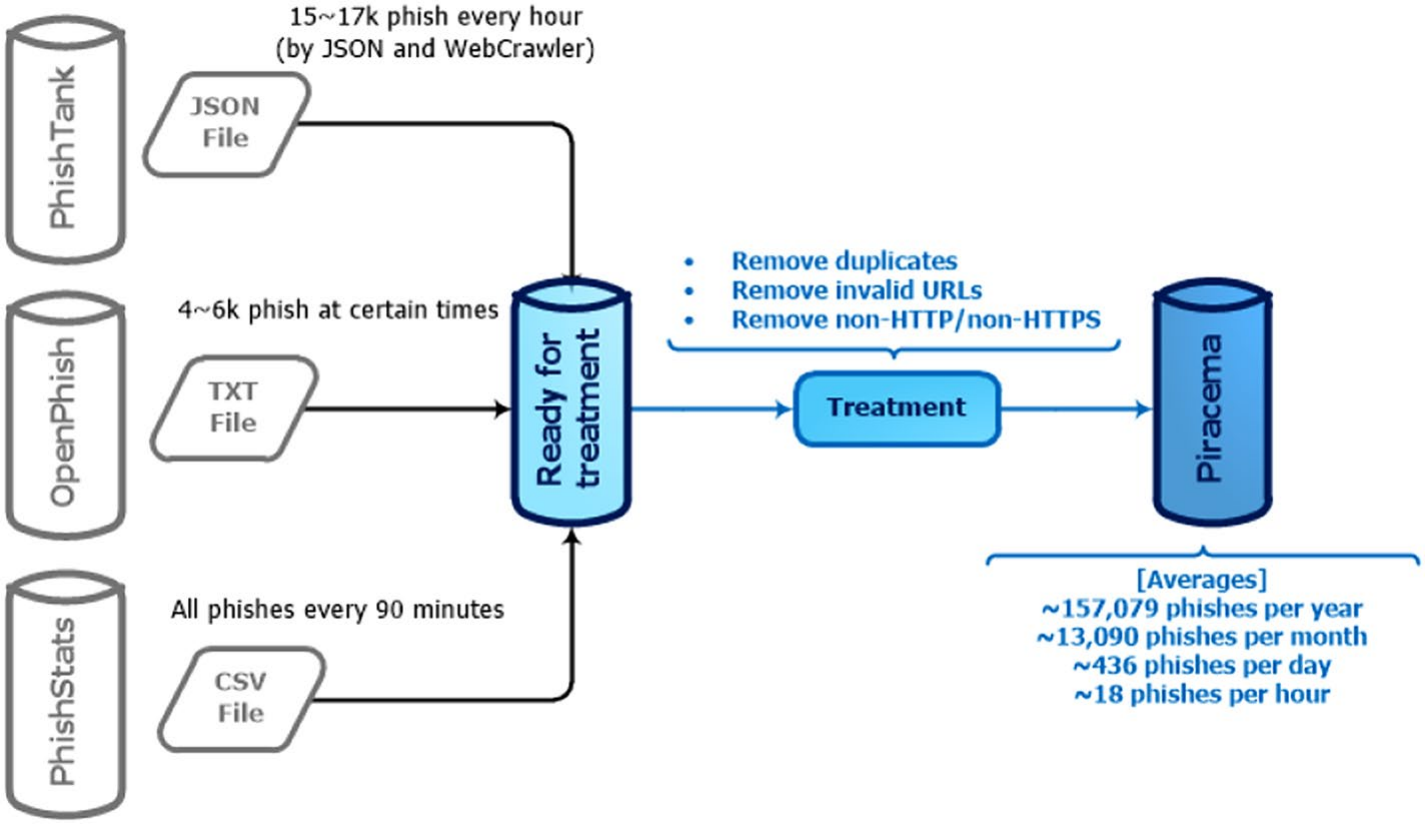
Extraction of phishing logs in each repository.

The database defined by this study, called Piracema, contains records of fraudulent pages extracted from 3 whistleblowing platforms that make their records available for free: PhishTank (https://phishtank.org/), OpenPhish (https://openphish.com/), and PhishStats (https://phishstats.info/). We note that each page on these platforms was reported by the community, analyzed, and received a verdict, judging whether it is phishing or a legitimate page. In addition, the pages in Piracema, dating from 2016 to 2021, were collected and organized by the reported year. Figure 1 illustrates the extraction process in each repository as well as the average number of records collected each period.

The building process of Piracema is detailed in the following sections: **Data Source**, where phishing reporting platforms are explained, from the suspicion of fraud to its confirmation. **Data extraction**, which aims to describe the data collection process on the whistleblower platforms. Moving on to **Data Treatment**, the improvements applied to the data will be exposed to maintain the base with consistent information. In **Features** are defined the extracted features from the data, their collection processes, improvements, and statistical data. **Subfeatures** exemplifies some implicitly features on the base, the result of a decomposition of the features presented in the previous section. Finally, **Threats** aims to present the found obstacles during the previous steps or possible limitations for the data use contained in the database.

### Data source

There are several platforms available that serve as support for phishing detection. In this study we used PhishTank, OpenPhish, and PhishStats, due to the greater diversity of records, availability and processed data.

**PhishTank** is a free community platform where anyone can submit, verify, track and share phishing data (https://bit.ly/39qG5bj). It also provides an open API to share your anti-phishing data with free third-party applications. It is important to point out that the PhishTank team does not consider the platform as a protective measure (https://bit.ly/3HpuYw0). For them, PhishTank information serve a subsidy for incident response mechanisms in various organizations (https://bit.ly/3MKJHCt), such as Yahoo!, McAfee, APWG, Mozilla, Kaspersky, Opera and Avira.

PhishTank is described as a **community** because it supports a large number of users who collaborate on phishing data on the Web. Its collaborative nature refers to the fact that all registered users have the possibility to feed the phishing database through voluntary reports.

**Figure 2:**
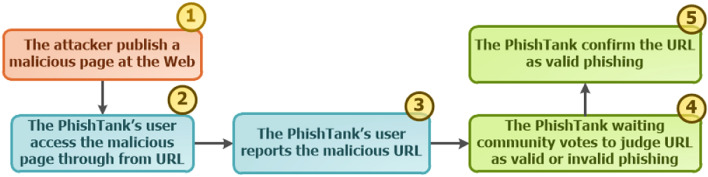
Lifecycle of the *PhishTank Community*.

Regarding confirmation, PhishTank allows a user to submit a suspicious URL and for other users to carry out a voting system to determine the verdict on the report, that is, to consider the phishing as valid or invalid. As for availability, the platform observes whether the phishing is online or offline. It is important to note that unavailable phishing means that the request returned HTTP code 400 or 500, that is, inaccessible, assuming the status “offline”. The lifecycle between phishing, the platform and its users is divided into 5 stages as illustrated in Fig. 2.

In step 1, the attacker publishes their malicious page on a web server, made available for propagation across the web. Step 2 forwards the discovery of the malicious URL by a user. Subsequently, they access PhishTank and report the URL, thus performing step 3. Step 4 describes the moment when the platform waits for community votes on the newly reported URL. Finally, step 5 occurs when the voting system receives a satisfactory amount to consider the URL malicious or not. It is worth noting that the “sufficient” amount of votes is not explained, the platform declares that it may vary according to the history of complaints (https://bit.ly/3tXnCub). In addition to this, due to its high dependence on the community, there is a delay on the part of the community regarding the confirmation of the reported complaint, between steps 3 and 5 (the difference between confirmation time and submission time), resulting in a temporal vulnerability window.

It is worth mentioning that the same process occurs analogously on the platforms OpenPhish and PhishStats.

### Data extraction

The records extraction, which was carried out by Silva et al.^18^ in their work, started in August 2018 and ended in January 2019, with data from 2016 to 2018. Records from 2019 to 2021 were obtained between 2020 and 2022, completing this database.

Some platforms, such as PhishTank, establish a voting criterion made by the community to confirm the reports they receive; in this way, it is possible to avoid the occurrence of false positives. However, as these platforms do not define a deadline for the voting verdict, it was necessary to adopt an interval margin for the extraction process. The collection was carried out following the metric of 1 month before the current month, i.e., the pool of January was closed until the last day of February. The unique requirement was the records had the submission date belonging to January. The extraction process followed successively for the other months.

To build Piracema, it was necessary to obtain a significant amount of phishing categorized as “valid”, whether online or offline. Taking PhishTank as an example, the platform provides a web service that provides a JSON file (https://bit.ly/3OfqTwi). It is updated every 1 h, containing approximately 15,000 records. In addition to the URL, status, confirmation, and publication date, the confirmation date and target are also available. The confirmation date refers to when the verdict appears (phishing or legitimate) for a URL. Since several organizations make simultaneous requests to the API, a system is adopted to avoid overloading the platform’s servers. Each request to the file is made with a key to identify the user. Then, this key is informed in the HTTP header with the limits and intervals of requests to be performed periodically (https://bit.ly/3aXnvrR). Currently, the platform does not provide registration for new keys.

**Figure 3:**
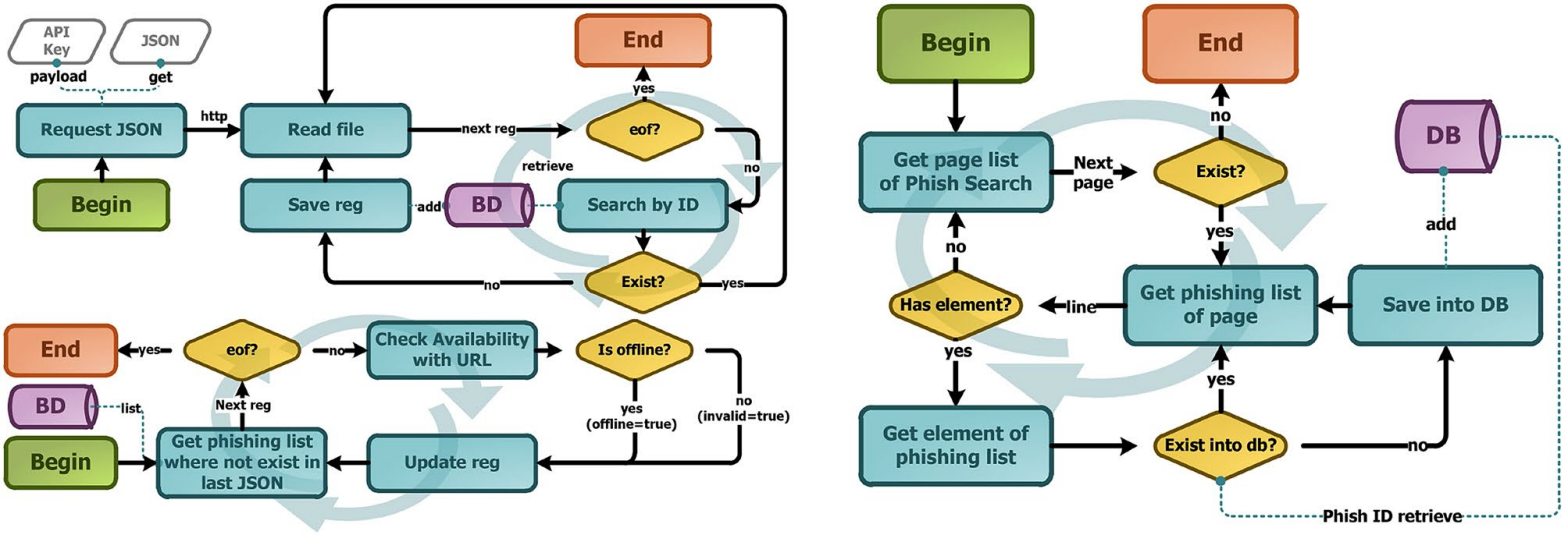
Extraction process flowchart.

However, the process had some obstacles. About 90% of the URLs were kept in the other subsequent files. Considering that each compressed JSON had 9MB, it overloaded the platform, giving a 509 bandwidth limit that exceeded errors, indicating that the key in the request had been banned. To circumvent this problem, previous registrations of several keys were performed to be replaced each time a key was banned, as shown in the flowchart on the left in Fig. 3.

Despite being rich in information, JSON has many limitations. One of these limitations is the option, on the part of the entity issuing the file, to keep only “valid” and “online” phishing records, thus disregarding temporal aspects that may impact the data. For example, this impact can be seen in the file downloaded on January 15, 2019, where the months of January and February 2018 had 358 and 617 records. However, the same file has more recent months, such as November and December of the same year, with 1524 and 1791 entries.

As an option to circumvent the limitations imposed by the JSON file, the platform offers the phish archive function (https://bit.ly/3Hqb2c7). Unfortunately, this functionality only allows the observation of information and not its download for storage and application in other activities. Therefore, several requests were made for access to the platform’s registry collection, but all attempts ended with no response from the PhishTank team. Therefore, with the lack of response from the platform, it was decided to develop a Web Crawler capable of collecting and storing the information available in the phish archive function. This process can be seen in Fig. 3.

On the other hand, JSON has a lot of useful information. For instanece, the “valid records” option allows to keep only phishing and “online” records, thus disregarding the temporal aspects that can impact data. This impact can be seen, for example, in the file downloaded on 01/15/2019, where the months of January and February 2018 had 358 and 617 records. However, the same file has more recent months, such as December of the same year, with 1524 and 1791 records for November.

As a service option provided as defined by the JSON file, a platform with phish file function (https://phishtank.org/developer_info.php). This feature of viewing, chronologically and through filters, of all URLs reported on the platform, that is, you can observe the platform records over time, still filtering as occurrences based on their validation status, “valid” or “invalid”, or by their availability of access, “online” or “offline”. This functionality only allows the observation of information, not its download for storage and application in other activities. Therefore, several requests for access to the platform collection were broadcast, but no attempt got response by PhishTank team. Thus, with the lack of response from the platform, it was decided to develop a Web Crawler capable of collecting and storing the information available in the phish archive function. This process can be seen in Fig. 3.

More efficient than JSON files, the data collection system through the Web crawler also faces some difficulties, such as the record list page, which limits the display of the long URL to the first 70 characters, abstracting the rest. Of the address and replacing it with “...”. As a workaround for this limitation, it was necessary to access the record’s detail page, where the web crawler could find the full URL version. At this point, it is essential to highlight that some entries were inaccessible on the details page, so, as a slight anomaly confined to several entries, it was decided to discard this data.

With the application of the web page collection mechanism, it was possible to extract the following information: record ID, URL, submission time, verification time (“valid” or “invalid”), and its availability (online or offline). This way, 942,471 records were obtained, spread over 6 years, from 2016 to 2021.

### Data processing

**Figure 4:**
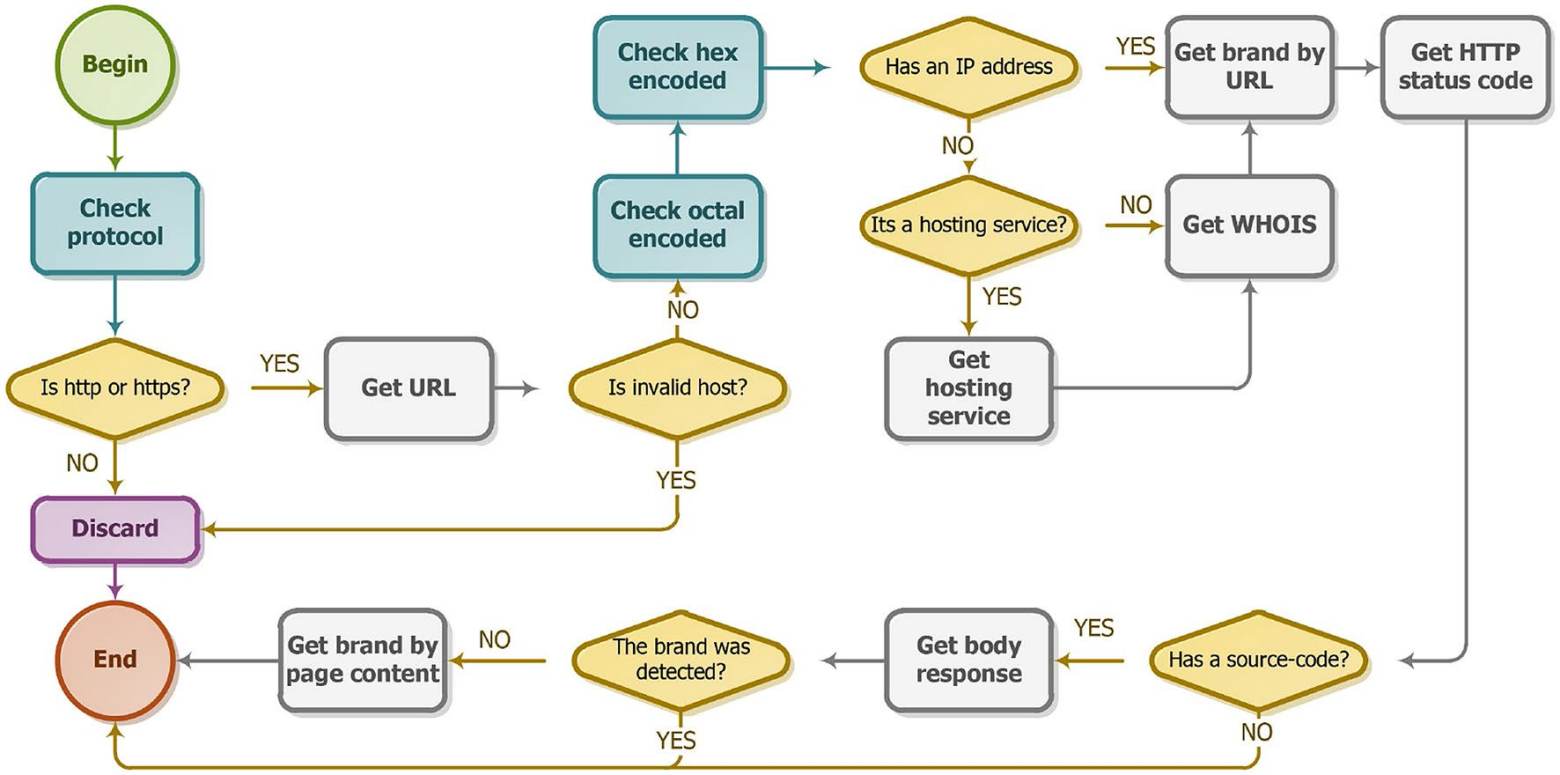
Flowchart of the treatment of records obtained.

The proposed consistent basis for its data refinement processes includes removing duplicates, removing false positives, and correcting inappropriate data. In this way, a more balanced basis is possible. During this process, as illustrated in Fig. 4, a refinement was obtained in the registered records, removing pages with an invalid host, motivated by the use of URLs with invalid syntax. The figure of the data performed, since information after an example cannot be previously or non-process obtained from the request, since information after a model cannot be obtained from the requirement of the process page has a registered domain, among other situations.

An analysis of the potential brands present in the sample was carried out to investigate the brands involved in the process. The PhishTank JSON has a “target” field. However, it was possible to observe that it has several records assigned as “Other”. In addition, it was also found that many values in this field did not match the target brand in question. In addition, the solution was to extract the marks involved through textual and visual search. Based on Table 10, it was possible to observe the most exploited brands in phishing attacks between the years 2016 to 2021.

### Features

**Figure 5:**
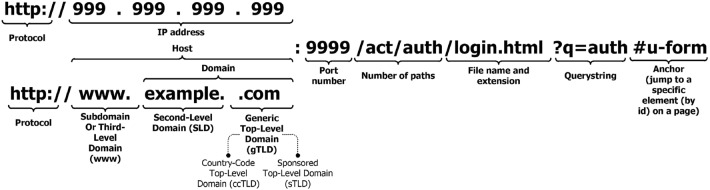
URL anatomy.

**Figure 6:**
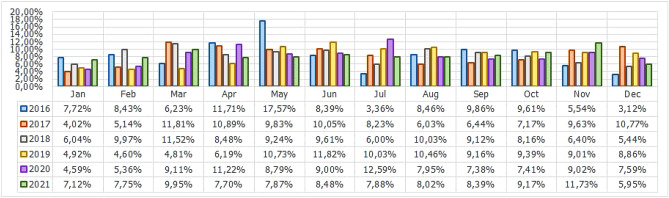
Phishing report by month over 2016 to 2021.

**Figure 7:**
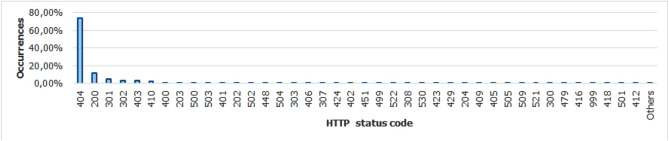
HTTP Status code occurences.

For the Piracema composition, each piece of information collected underwent individual pre-processing, which varied according to the nature of the data. In this process, several manipulations were performed, providing a refinement of the worked features. The description, relevance, and improvement applied to each feature are described in the following items:

- URL: This is the URL of the fraudulent page. This feature is the starting point for the other. We suggest the idea of analyzing the sub-features arising from it, such as the most explored TLD, URL size, number of subdomains, domain size, URL path analysis, if the port is different from 80, if the IP is exposed or not, among others, according to the anatomy illustrated in Fig. 5. This data was extracted from phishing repositories and invalid, duplicate, and notoriously false positive URLs were removed.
- Report Time: This field is the date the phishing was confirmed as a fraud by the repository. According to the time of year, it is possible to notice an increase in the number of cases of phishing attacks, motivated, for example, by the approaching a commemorative date, such as Christmas, or even promotional events, such as Black Friday, or even the pandemic caused by the Coronavirus. From the phishing Report Time, it is possible to analyze the seasonality and volatility of attacks (assuming that the date in question begins the phishing period). This information could be extracted from phishing repositories, where it did not need treatment, as illustrated in Fig. 6.
- Status Code: is the HTTP code of the page, which allows us to know which pages have a code of the family 200, 300, 400, or 500, with the response code 400 being the most present among the records since phishing pages tend to be highly volatile, with a very small period of activity. The extraction of this feature was performed via a Python script and did not need any specific treatment. The codes most found during the analysis of fraudulent pages are described in Tables 1 and 2, where the Status Code number is available, followed by the number of occurrences each year (2016-2021), the total sum of occurrences over the analyzed period and finally the percentage that this value represents about the total records. For more easily, this information can also be visualized in Fig. 7 and the Tables 1 and 2.

**Table 1:** Status code occurences #1.

# Status code	2016	2017	2018	2019	2020	2021	Total	%
404	59,695	66,236	134,109	128,682	160,050	185,152	733,924	73.47
200	3078	14,011	20,890	8464	12,754	54,014	113,211	11.33
301	6528	6042	10,933	12,657	11,265	333	47,758	4.78
302	2548	5098	9791	5947	8018	159	31,561	3.16
403	2211	2413	5672	7331	11,657	751	30,035	3.01
410	86	1101	2776	9864	9927	448	24,202	2.42
400	654	456	613	680	1758	529	4690	0.47
203	56	520	3351	100	171	20	4218	0.42
500	220	288	527	937	951	565	3488	0.35
503	92	109	269	448	931	46	1895	0.19
401	36	47	198	161	301	17	760	0.08
202	13	0	3	275	161	1	453	0.05
502	66	66	169	48	54	4	407	0.04
448	4	36	174	38	53	19	324	0.03

**Table 2:** Status code occurences #2.

# Status code	2016	2017	2018	2019	2020	2021	Total	%
504	0	6	6	1	3	282	298	0.03
303	20	36	68	61	43	2	230	0.02
406	21	34	61	21	73	0	210	0.02
307	15	31	53	52	49	5	205	0.02
424	1	7	31	100	51	2	192	0.02
402	2	6	23	34	86	10	161	0.02
451	0	1	4	35	68	10	118	0.01
499	0	30	82	0	0	0	112	0.01
522	0	0	0	0	0	107	107	0.01
308	9	1	1	21	27	5	64	0.01
530	0	7	6	1	35	0	49	0.005
423	0	2	3	26	4	1	36	0.004
429	1	0	2	8	15	5	31	0.003
204	0	4	15	4	1	0	24	0.002
409	1	4	4	10	2	0	21	0.002
405	0	4	12	1	3	0	20	0.002
505	13	1	4	0	0	0	18	0.002
509	3	0	4	0	4	0	11	0.001
521	0	0	0	0	0	8	8	0.001
300	1	2	2	0	1	0	6	0.001
479	1	3	1	1	0	0	6	0.001
416	0	0	0	1	4	0	5	0.001
999	0	1	0	0	0	3	4	0.0004
418	2	1	0	0	0	0	3	0.0003
501	1	0	2	0	0	0	3	0.0003
412	0	0	2	0	0	0	2	0.0002
Others								0.001

**Figure 8:**
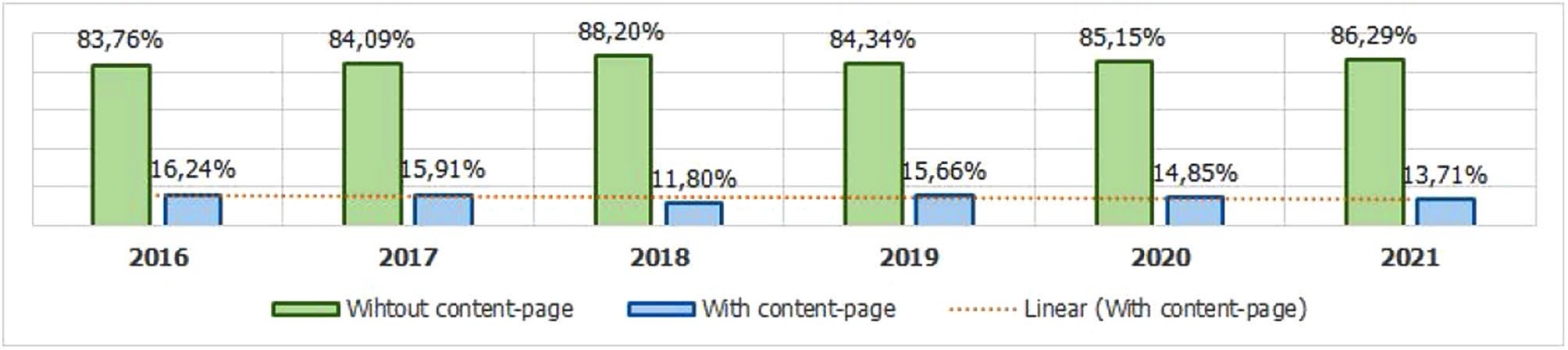
Phishing with and without content-page over 2016 to 2021.

**Figure 9:**
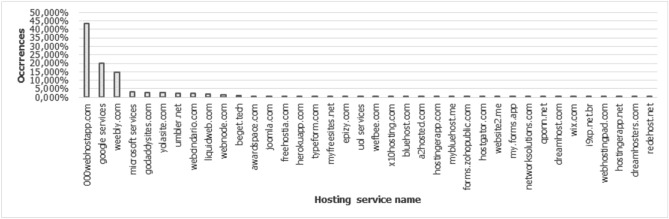
Hosting services involved in the evaluation process.

- Response content: As illustrated in Fig. 8, this field refers to the content of the page body (HTML, CSS and JS source code). The source code can help in analyzing the behavior of a malicious page, resulting in subfeatures, as it happens when analyzing the URL. In case the content of the source code is significant, you can analyze features such as cross-domain forwarding and clickjacking attempts and fake user errors^25^. This last feature is present in attacks aimed at mobile devices, where the fraud is developed according to the devices resolution and if the user tries to open the link through a desktop browser, an HTTP error is displayed that was simulated by the attacker^18^.
- Hosting service: Defines whether the page in question is hosted on a hosting service. This kind of information is important because pages of this nature do not have a registered domain, therefore, it will not be possible to detect their age through WHOIS. In addition, it is possible to analyze the hosting services most exploited for crime. This type of service can generate a series of benefits for the attacker, enhancing the publication of fraud. We can cite as an example 000webhost, which offers free and easy-to-use hosting, reason that leads it to be among the most used hosting services in phishing attacks, as seen in Fig. 9 and in more detail in Table 3. It can also be seen in Table 3 the presence of hostings with the name of **Google services** and **Microsoft services**; these items refer to services made available by **Google**, such as: *blogspot.be*, *blogspot.com*, *docs.google.com*, *drive.google.com*, *firebaseapp.com*, *forms.gle*, *googleapis.com*, *sites.google.com* and *web.app*. The term **Microsoft services** is the grouping of services as: *myspace*, *office.com*, *onedrive.live.com* and *sharepoint.com*. Therefore, all these services were considered as a single item associated with the company to which it belongs. The data was extracted via Python script, with NLP and Regex resources. Prior to the extraction, APWG reports were consulted to identify the most exploited hosting services in phishing attacks between 2016 and 2021. Next, the data were used as a knowledge base to carry out the detection.

**Table 3:** Hosting services involved in the evaluation process.

#	Hosting service	2016	2017	2018	2019	2020	2021	Total	%
1	000webhostapp.com	10	665	2490	10,354	10,668	8698	32,885	43.26
2	google	99	294	818	3093	5296	5661	15,261	20.08
3	weebly.com	14	244	535	462	3036	6764	11,055	14.54
4	microsoft	37	44	132	1341	794	181	2529	3.33
5	godaddysites.com	0	0	1323	308	400	183	2214	2.91
6	yolasite.com	11	44	89	62	407	1522	2135	2.81
7	umbler.net	0	9	106	855	633	100	1703	2.24
8	webcindario.com	37	41	225	657	377	255	1592	2.09
9	liquidweb.com	166	196	352	233	271	268	1486	1.95
10	webnode.com	4	16	220	206	479	124	1049	1.38
11	beget.tech	0	139	319	204	171	7	840	1.11
12	awardspace.com	66	18	460	0	0	0	544	0.72
13	joomla.com	0	0	17	184	278	2	481	0.63
14	freehostia.com	56	66	224	3	0	0	349	0.46
15	herokuapp.com	6	4	19	42	39	229	339	0.45
16	typeform.com	0	8	13	99	167	50	337	0.44
17	myfreesites.net	16	104	141	4	6	6	277	0.36
18	epizy.com	1	8	54	39	93	7	202	0.27
19	uol	21	8	77	0	0	0	106	0.14
20	wefbee.com		0	84	0	0	0	84	0.11
21	Others								0.72

**Figure 10:**
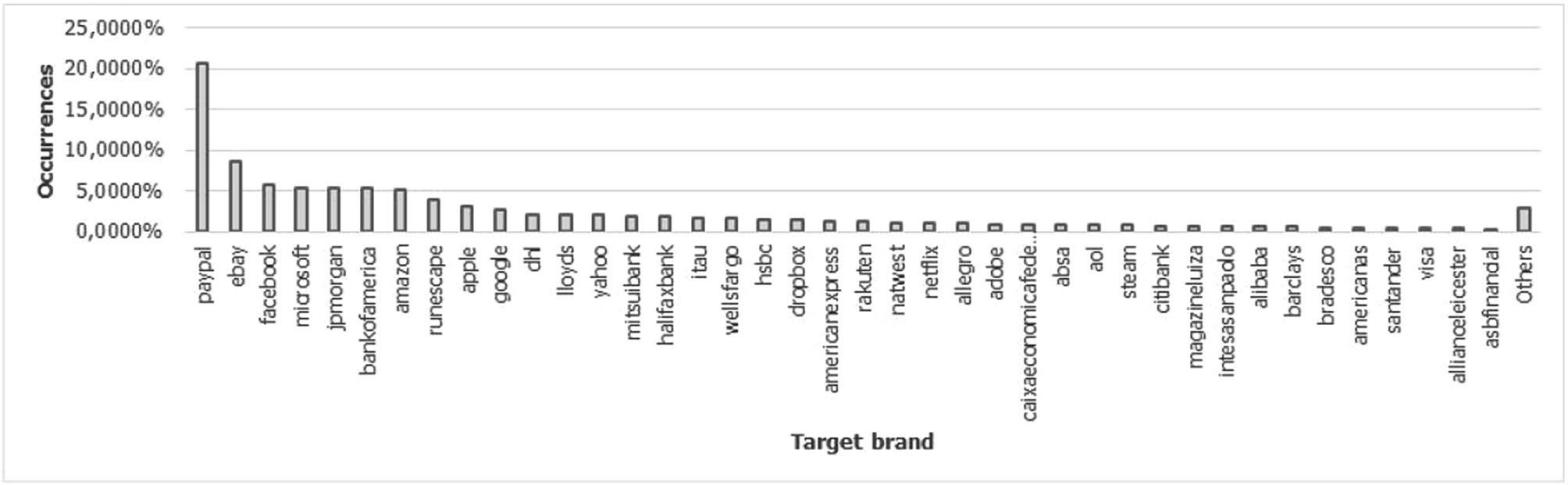
Brands involved in the evaluation process.

- Target brand: It is the target brand identified in the fraud; its identification can be an important premise in preventing the phishing attack, especially in targeted attacks, which are usually very sensitive to aspects of the visual identity of a particular brand. It is possible to consider that certain elements offer greater trustworthiness to the attack, increasing its effectiveness. Given this, through social engineering, the attacker observes visual aspects of the content, context and URL of the page. The whole motivation for this effort, on the part of the attacker, is to create a greater susceptibility of the end user to phishing attacks^18^. It was possible to extract this feature using a Python script, supported by NLP and Regex. Before starting to extract the feature, we used APWG reports, identifying the most exploited brands in phishing attacks between 2016 and 2021. Then, we created a list with these marks, using as a base of previous knowledge for the detection algorithm. To start the brand detection, the hosting service detection was previously performed, that is, if the page in question was hosted on a hosting service. For example, in the link https://sites.google.com/s/paypal-secure-access, as it was previously detected that it is a google hosting service, the detection mechanism discards the keyword check on the domain, focusing only on the subdomain and URL path, avoiding false positives in relation to the target brand. The most common marks in phishing attacks are illustrated in Fig. 10 and have their detailed information in the Table 4.

**Table 4:** Top 20 brands involved in the evaluation process.

#	Brand	Year							
–		2016	2017	2018	2019	2020	2021	Total	%
1	paypal	8493	10,174	20,953	8921	4704	1884	55,129	20.71
2	ebay	3870	6065	9906	581	1457	922	22,801	8.57
3	facebook	470	874	1252	2008	4491	5969	15,064	5.66
4	microsoft	532	603	1726	4171	5161	2235	14,428	5.42
5	jpmorgan	1614	632	9936	508	1154	325	14,169	5.32
6	bankofamerica	1641	1446	5447	3814	1493	95	13,936	5.24
7	amazon	113	203	193	1397	3964	7835	13,705	5.15
8	runescape	6	13	119	3105	4659	2388	10,290	3.87
9	apple	552	353	623	3972	1985	688	8173	3.07
10	google	590	540	2205	839	2123	672	6969	2.62
11	dhl	214	268	454	1565	1564	1570	5635	2.12
12	lloyds	237	120	1074	23	1740	2415	5609	2.11
13	yahoo	324	400	935	2015	1060	757	5491	2.06
14	mitsuibank	35	4	9	393	1305	3455	5201	1.95
15	halifaxbank	306	298	755	3	3391	364	5117	1.92
16	itau	47	83	619	764	2551	556	4620	1.74
17	wellsfargo	253	291	410	663	1415	1516	4548	1.71
18	hsbc	613	677	1431	69	397	670	3857	1.45
19	dropbox	766	500	443	1053	638	289	3689	1.39
20	americanexpress	308	113	1735	441	583	312	3492	1.31
21	Others								16.63

**Figure 11:**
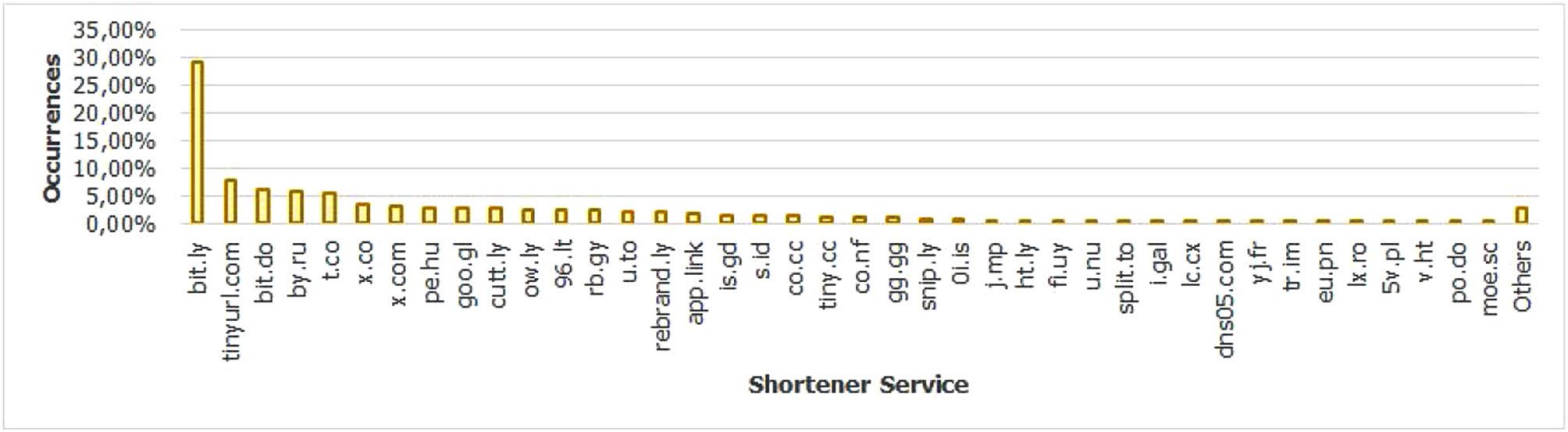
Shortener services involved in the evaluation process.

- Shortener Service: Defines if the page has the use of a URL shortener service, where a website has its URL converted into a short URL code. This type of service is widely used in an attempt to hide features of the fraudulent URL, leading the end user to access the page in question, since it is not possible to analyze in advance aspects of the URL such as the domain name. The most commonly used URL shortening services in phishing attacks are shown in Fig. 11 and detailed in Table 5. The data was extracted via Python script, using NLP and Regex techniques. A survey was done previously, looking for the most used shorteners on the web, followed by APWG reports to filter the most exploited shorteners in phishing attacks between 2016 and 2021. In order to cover as many records as possible, services with lesser popularity were added, searching for domains with a length of less than 5 characters, as well as domains whose host and domain were the same and there was no subdomain. Finally, we used all occurrences as a knowledge base to carry out the detection.

**Table 5:** Top 20 shortener services involved in the evaluation process.

#	Shortener service	2016	2017	2018	2019	2020	2021	Total	%
1	bit.ly	202	625	787	700	830	1798	4942	29.35
2	tinyurl.com	186	249	343	220	156	149	1303	7.74
3	bit.do	6	40	199	147	354	309	1055	6.27
4	by.ru	189	154	631	0	0	0	974	5.78
5	t.co	20	41	252	104	136	342	895	5.32
6	x.co	7	27	215	311	2	0	562	3.34
7	x.com	0	1	215	311	2	0	529	3.14
8	pe.hu	79	164	230	16	2	0	491	2.92
9	goo.gl	39	125	307	14	1	0	486	2.89
10	cutt.ly	0	0	1	38	76	339	454	2.7
11	ow.ly	89	104	182	18	37	11	441	2.62
12	96.lt	22	190	198	14	1	0	425	2.52
13	rb.gy	0	0	0	0	41	370	411	2.44
14	u.to	3	28	28	118	58	118	353	2.1
15	rebrand.ly	0	3	30	59	138	121	351	2.08
16	app.link	0	2	2	101	166	57	328	1.95
17	is.gd	3	6	27	69	80	81	266	1.58
18	s.id	0	2	6	31	44	171	254	1.51
19	co.cc	49	49	133	0	0	0	231	1.37
20	tiny.cc	28	19	84	65	17	2	215	1.28
21	Others								11.12

- WHOIS: It is the creation date of the domain, important to know the age of the domain that was registered; this information is useful for analyzing phishing volatility patterns. It was extracted via a python script, looking only for records where there is no page on hosting services, thus avoiding data coming from a domain registered by the owner of the hosting service and not something registered by the malicious person for their fraudulent page.

#### Subfeatures

From the analysis of the features presented in the base, it is possible to observe a significant variety of secondary features, such as **domain size** and **number of subdomains**, which can be extracted from the URLs, or the **time of activity** of the fraudulent pages, derived from the relationship between the date of registration of the domain and the date of its identification. Such features, created from the decomposition of larger features, are called subfeatures. These features can be extremely valuable for application in ML models, since they can help in the detection of behavioral patterns, capable of identifying the perversity of fraudulent pages.

Additionally, it is possible to link these subfeatures to certain phishing behaviors, whose some aspects are debated in the literature, such as spread and volatility. Based on studies by Silva et al.^17,18^, see the nomenclatures and definitions adopted by this study to explain the behaviors commonly observed in phishing:

- **Trustworthiness**, describes the high richness of fraud details compared to the genuine page. In view of this, the attacker extracts profiles of each target involved, which translate into a set of behaviors that serve as a subsidy for the elaboration of the malicious page^26^. In theory, the higher the quality of the profile, the greater the trustworthiness. Also, the attacker can also carry out other activities, such as registering or hijacking a domain, in order to assign arbitrary combinations through keywords. This behavior is also sensitive in the detection of brands involved in the process, distinguishing conventional phishing from targeted phishing^18^.
- **Obfuscation**, which describes the fraudster’s attempts to hide information that could be visible to the end user, but due to high or low amount of characters, some details may not be observed. It is not uncommon for malicious actors to apply techniques that forge behavior through JavaScript, simulating errors or restrictions in order to target their attacks to a particular region or device.
- **Propagation**, which describes some behaviors that aim to increase the reach of frauds on a large number of users, such as bypassing techniques in blocking lists. In the same vein, the exploitation of services on the Web, such as hosting and domain registration services, end up driving the spread of fraud.
- **Seasonality**, which describes the sensitivity of phishing to annual calendar events. Interestingly, in Fig. 19, about the Data Records section, an apparently stable pattern of occurrences between the months of August and October is noticeable, as well as a peak pattern in the months of November in the last 6 years. On the other hand, in this same annual window, it is possible to observe outliers, one higher case in July 2020 and two lower cases in the same month in 2016 and 2018, such facts can be justified by some seasonal event. It is important to analyze these outliers, as data that differs drastically from all others can cause anomalies in systems that analyze patterns of behavior.
- **Volatility**, which refers to the short lifespan, showing that the fraud is quickly abandoned by its creator. Volatility can be an obstacle in the studies of phishing behavior, since much evidence of fraud can be analyzed in the source code, a resource of which ends up being available for a very imminent time.

In Tables 6 and 7, some subfeatures are named and briefly defined, followed by the behaviors to which they can be related, the type of the variable and from which main feature they can be collected. These subfeatures are implicitly at the base, explained in this section because they came from our analysis, which made it possible to glimpse them. But the number of possible subfeatures is not restricted to the ones mentioned above, since analyzes by other authors may result in new subfeatures.

**Table 6:** Subeatures Definition #1.

#	Subfeature	Description	Related behaviors	Type	Feature collect
1	Host	Hosting service exploited by attackers to publish their scam on the web. Due to the existence of free and easy-to-use services of this type, the fraudster can leverage the publication of their fake pages	URL	Text	URL
2	Domain	Term used in name service (DNS)	URL	Text	URL
3	Subdomains	Secondary address linked to primary domain. Considering that the URL address bar has a limited size, it is not uncommon for fake pages to use multiple subdomains in an attempt to mask the main domain name	URL	Text	URL
4	Port	Port used to access the machine where the service is hosted. The most used in fraud are available in Fig. 13	URL	Number	URL
5	Path	URL string that corresponds to the domain (or port) after the last slash “/”, as it has an arbitrary value, it is taken as a variable	URL	Text	URL
6	Querystring	String of the URL that follows the path, starting with a “?”, as it has an arbitrary value, it is taken as a variable	URL	Text	URL
7	Certificate	Page with active digital certificate. More and more attackers are valuing visual richness in their frauds, in order to forge its trustworthiness, so a page with an active security certificate creates greater susceptibility of the end user	URL	Boolean	URL
8	Ip address explosure	Situations where the host does not have a DNS, and the IP of the web-published machine is displayed. In these cases, the user is not able to evaluate the URL’s features in advance	URL	Boolean	URL
9	Path with keywords	Application of specific keywords in the URL path	URL, Trustworthiness	Text	URL
10	Url-based brand detection	Target branding in parts of the URL. As a way to create greater user susceptibility, it is common for the attacker to use words that are related to the target brand of the attack in the page URL	URL, Trustworthiness, Target Brand	Text	URL/Target brand
11	Look-alike trust domain	Domain with relevant similarity to legitimate domains	URL, Trustworthiness, Target Brand, Obfuscation	Text	URL/Target brand
12	Domain length	Number of characters in the page domain. The occurrences extracted from the base are available in Fig. 17	URL, Obfuscation	Number	URL
13	Url size	Number of characters in the URL. The occurrences collected in the base can be seen in Fig. 14	URL, Obfuscation	Number	URL
14	Ip address enconded	IP obfuscated by some kind of encoding, such as hex, octal or punycode, these cases were observed in the base, as illustrated in Fig. 12	URL, Obfuscation, Propagation	Boolean	URL
15	Number of subdomains	Number of subdomains applied to the URL. The occurrences extracted from the base are available in Fig. 18	URL, Obfuscation	Number	URL
16	Number of paths	Number of paths defined in the URL. The occurrences collected in the base can be seen in Fig. 15	URL, Obfuscation	Number	URL
17	Tld most exploit	Most exploited domain registration, as seen in Fig. 16	URL, Trustworthiness, Propagation	Text	URL
18	Number of separators	Number of separators applied to the URL	URL, Obfuscation, Propagation	Number	URL
19	Spoofed URL	Pattern of using separators in the URL, to result in apparently safe or legitimate URLs, in order to deceive victims	URL, Obfuscation, Propagation	Text	URL
20	Shortener URL	URL shortener service exploited by attackers to publish their scam on the web	URL, Obfuscation, Propagation, Shortener Service	Text	URL/Shortener service
21	Url-variable exploit for bypass	Purposeful variations in the URL path and/or querystrings in order to “bypass” blacklist mechanisms, since any changes to these variables end up changing the generated hash	URL, Propagation	Text	URL
22	Url-based seasonal keywords	Application of words that refer to the temporal context experienced at the time of publication of the page	URL, Target Brand, Trustworthiness, Seasonality	Text	URL/Target brand

**Table 7:** Subeatures Definition #2.

#	Subfeature	Description	Related behaviors	Type	Feature collect
23	Period most exploited	Times of the year when the number of phishing attacks are most exploited	Report Time, Propagation	DateTime	Report time
24	Exploits in periodical events	It seeks to identify patterns of occurrences of attacks in certain seasonal periods of the calendar year	Report Time, Seasonality	DateTime	Report time
25	Exploits in non-periodical events	It seeks to identify attacks that occur in less prone seasonal periods (outliers)	Report Time, Seasonality	DateTime	Report time
26	Community report delay	Latency period between the publication of the page and its identification (final verdict) as malicious	Report Time, Volatility	DateTime	Report time
27	Status code forgery	Forging user-facing errors, where the fraud is developed considering the device’s screen resolution, very common in mobile device attacks. In this scenario, if the user tries to access the page through a desktop browser, an error is displayed, simulated by the attacker, asking the user to access the link through a cell phone	Status Code, Obfuscation, Propagation	Text	Http status code
28	Status code life-cycle	Length of time the page returns a given response code	Status Code, Volatility	Number	Http status code
29	Title page	Page title, extracted from the content of the html title tag	Response content	Text	Response content
30	Meta description	Description of the page, extracted through the html content of the meta description with name equal to description	Response content	Text	Response content
31	Content-based brand detection	Identification of the target brand in parts of the web page content	Response content, Trustworthiness	Text	Response content
32	Targeted phishing	Targeted phishing, that is, with a wealth of details directed at a particular target brand	Response content, Trustworthiness, Obfuscation	Boolean	Response content
33	Device-based behavior forgery	Frauds aimed at the mobile environment, present forged behavior when accessed on devices with different screen resolution	Response content, Obfuscation	Boolean	Response content
34	Malicious redirections	Exploitation of redirection flaws (Cross-Site Request Forgery, CSRF) on legitimate pages, which end up redirecting the user to environments outside their domain and possibly hostile	Response content, Obfuscation, Propagation	Text	Response content
35	Cloning detection	From the analysis of the page content, it is possible to collide the response content hash to verify that the pages are identical	Response content, Propagation	Boolean	Response content
36	Language most exploited	Language most exploited by malicious people	Response content, Propagation	Text	Response content
37	Content-based seasonal keywords	Detection of keywords that refer to commemorative periods, such as Christmas, Black Friday and so on	Response content, Propagation, Seasonality	Text	Response content
38	Service most exploited	Most exploited services on malicious pages	Hosting Service, Propagation	Text	Hosting service, Shorterner service
39	Service detection delay	Time between the fake page being created and the reporting and blacklisting process	Hosting Service, Volatility	Text	Hosting service, Shortener URL
40	Look-alike targeted domain	Malicious domains forged with high trustworthiness, cases extensively exploited in cybersquatting and typosquatting^18,27^	Target Brand, Trustworthiness, Obfuscation	Boolean	Target brand
41	Segment-type most exploited	Niches of services most exploited in malicious attacks, such as e-commerce, social networks, financial transactions and so on	Target Brand, Propagation, Seasonality	Text	Target brand
42	Brand most exploit	Certain brand more prone to attacks	Target Brand, Trustworthiness, Seasonality, Volatility	Text	Target brand
43	Seasonal terms for brand	Presence of seasonal terms targeting the target brand such as “Day Amazon”	Target Brand, Trustworthiness, Seasonality, Volatility	Text	Target brand
44	Age of domain	Domain uptime observed from the difference between its registration date and the time of its inactivity	WHOIS Creation Time	Number	Whois creation time
45	Phishing activity	Uptime that the phishing attack remains active	WHOIS Creation Time, Volatility	Boolean	Whois creation time

**Figure 12:**
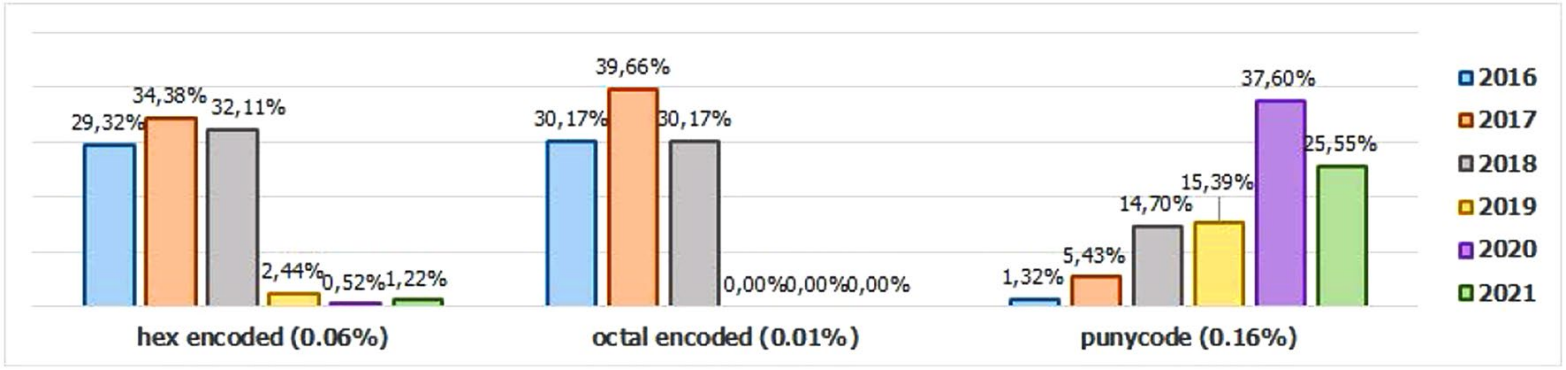
IP address encoded occurences.

**Figure 13:**
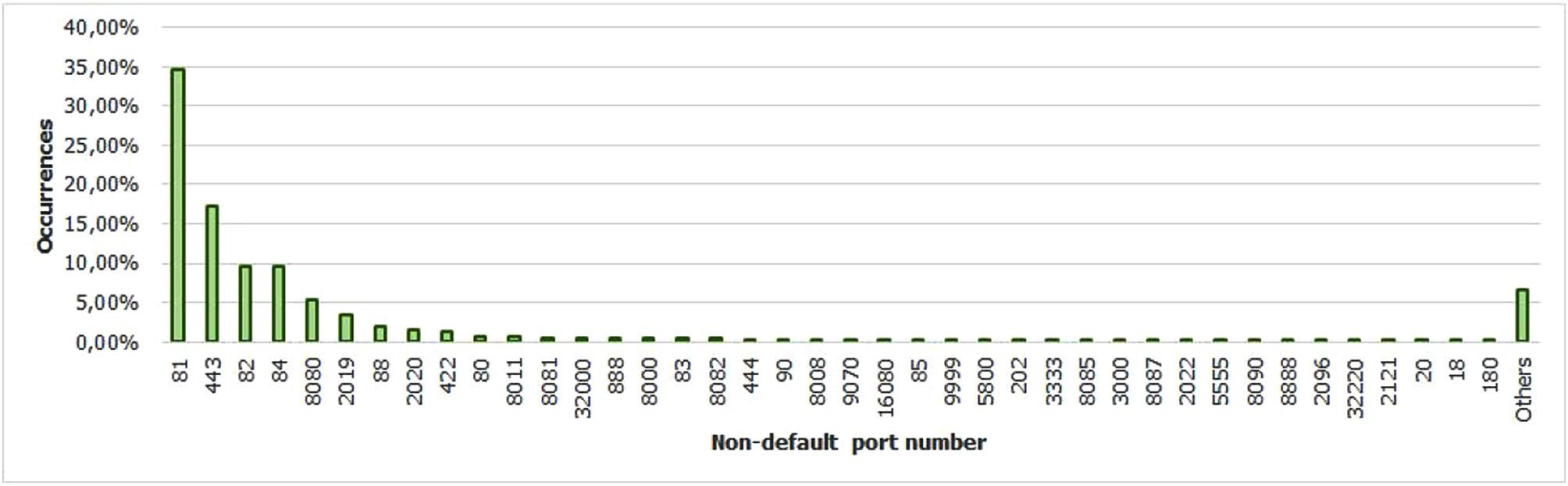
Non-default port number occurences.

**Figure 14:**
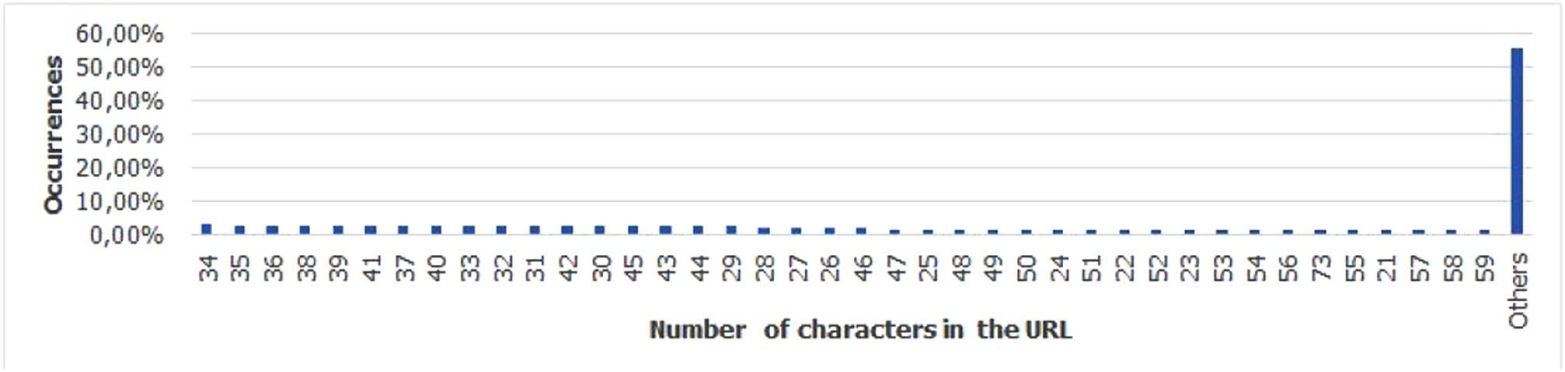
URL size occurences.

**Figure 15:**
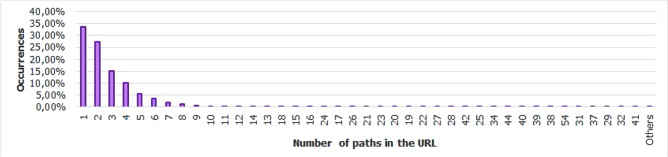
Number of paths occurences.

**Figure 16:**
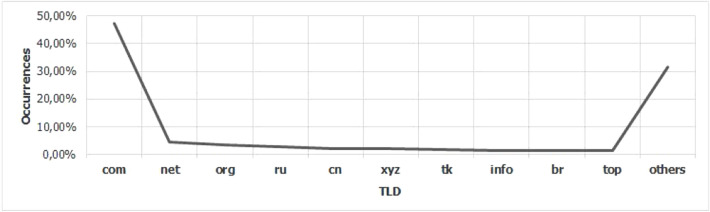
TLD most exploit analysis.

**Figure 17:**
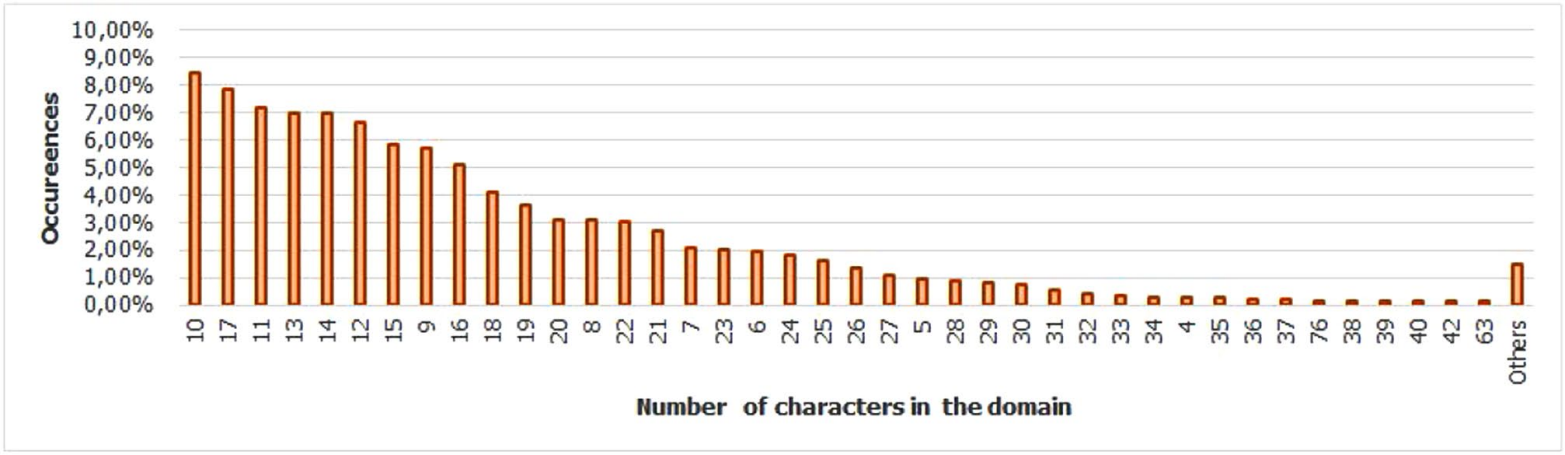
Domain length occurences.

**Figure 18:**
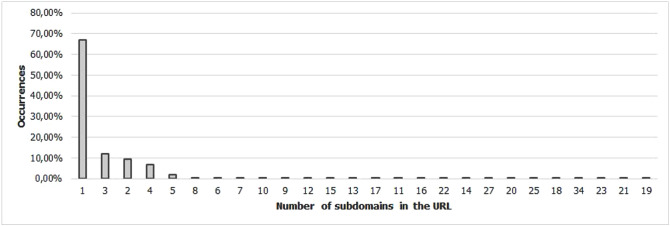
Number of subdomains occurences.

### Threats

This section describes the threats and barriers to be considered by the study.

Regarding the sample collection process, it was not uncommon to find duplicate occurrences between the 3 reporting platforms, so the final amount of fraudulent pages to be analyzed dropped significantly. Although 8 features considered relevant were gathered, a significantly large amount for a single database, some important features may have been left out.

When capturing the Report Time of a phishing page, there may be cases where the phishing activity did not necessarily start in the informed period (the same may have acted much earlier, as there may be a delay in the community report).

Since the extractions started between 2019 and 2020, when we gathered the Status Code of the pages, the predecessor years end by having fewer records showing code 200 errors or similar, due to the volatility of phishing. To do so, the process was extracted via a python script, then content that offered an error page from the hosting service was removed, where the page had been removed and only a standard redirect warning was displayed. The entire process was supported by the Status Code information, as can be seen in Table 8. Similarly, the Response feature ends up being affected by the volatility of phishing, so that in recent years, the number of pages with the source code ends up being higher.

**Table 8:** HTTP Status code parsed for extracting page content.

Code	Description	Extracted content?	Justification (if not extracted)
200	OK	Yes	–
202	Accepted	Yes	–
203	Non-Authoritative Information	No	Do redirect
204	No content	No	Empty
300–399	Redirection types	No	A standard hosting message is displayed in the body of the page
400–499	Client error types	No	A standard client-side message is displayed in the browser
500–599	Server error types	No	A standard client-side message is displayed in the browser

Hosting services and URL shorteners most used in attacks were detected. However, there may be other services that are not on our prior knowledge list that could be used to host malicious pages. The high volatility of the phishing scenario causes hosting service maintainers to have a considerable delay in identifying the use of their services for fraudulent purposes (that is, when they do), causing the malicious user to keep migrating from service each time it is banned. Such a delay can also end up making the conduct policy of these services unfeasible, motivating fraudsters to increasingly explore a particular service. Similarly, the target brand is also detected by a list of prior knowledge generated from the latest reports from the APWG, then the detection engine may experience the same overfitting mentioned earlier.

Finally, in the WHOIS capture process, the limitation refers to the fact that the information base of domain registrars is is limited to .COM, .NET, .EDU. Another point worth mentioning are the cases of phishing attacks that hijack legitimate domains, such as cases when a malicious person manages to inject a malicious page through exploitation via upload on a legitimate domain with a registered domain. In these cases, for the most part, they were removed by the refinement process because they were assumed to be false positives, considering that, once the server maintainer removes the malicious file, your domain will no longer be dangerous.

However, in some situations, such as the case of overfitting due to absence from the previous list, the WHOIS may end up resulting in a very old activity date (because it is a hijacked legitimate domain), which would bias the results, however, we believe that there are few cases of this nature, since we performed a screening in search of significant outliers.

## Data records

This section describes the contents present in each database file. As shown in Table 9, the base keeps files divided by year, individually, with phishing page data from 2016 to 2021, followed by the number of occurrences for each year. In Table 10 it is possible to observe the extracted features and their descriptions.

**Figure 19:**
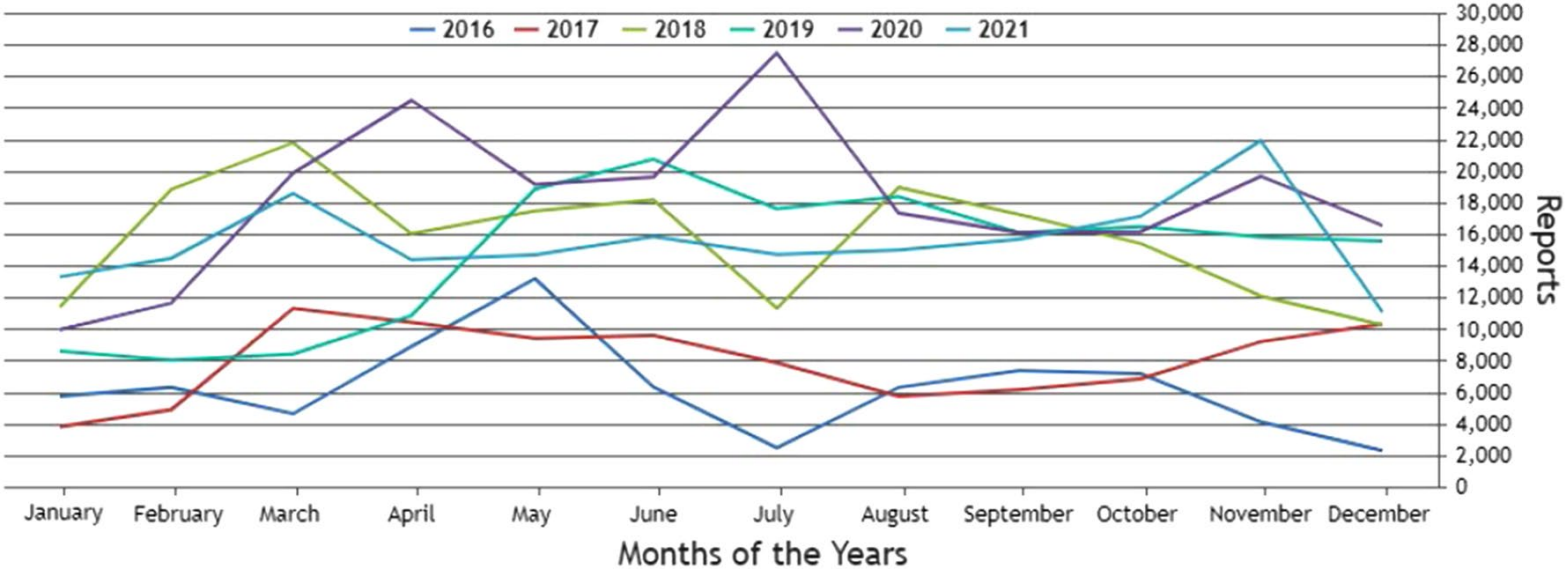
Summary of phishing reports over the 2016 to 2021.

**Table 9:** Entries content details.

Year	All	With content	Hosting service detected	Target brand detected	Shortener service detected	Whois creation date extracted
2016	75,130	12,202	572	24,441	1050	–
2017	96,195	15,307	1971	28,887	2128	–
2018	189,491	22,355	7809	66,854	4025	–
2019	175,976	27,566	18,294	44,405	2296	–
2020	218,459	32,441	23,167	57,505	2430	–
2021	187,220	25,671	18,784	36,321	3079	123,285
Total	942,471	135,542	70,597	258,416	15,008	123,285

**Table 10:** Entries structure details.

Fied	Type	Description
url	LONGTEXT	URL’s page
report_time	DATETIME	Timestamp during phishing report/catch
http_status_code	INT	HTTP Status code during on the content-page extraction
response_content	LONGTEXT	HTTP Body content of the maliciou’s page
hosting_service	VARCHAR	Cases of the malicious page hosted on hosting service and recognized based on Regex and NLP. This detection based on URL; when these entries with the registered domain have been discarded.
target_brand	VARCHAR	Target brand recognized based on Regex and NLP
shortener_service	VARCHAR	URL shortening service recognized based on Regex and NLP
whois_creation_date	BIGINT	The domain age based on WHOIS lookup result (applied only .com, .net, and .edu registered domains)

## Technical validation

The main contributions proposed by the Piracema database are highlighted in Table 11, including comparisons of some features present in the PhishTank, OpenPhish, and PhishStats repositories. Note that some items in Table are marked with “*” to represent some reservations about how the content is obtained or presented in the base to which it is related.

**Table 11:** Comparison between the databases involved in the study.

Platform name	Does the platform collect user reports about new phishing?	Data offered	Pre-processing approach	Does the platform available an API?
PhishTank	YES	URL; Target; submit_time	Analyzes the entries as “valid” or “invalid”*; Analyzes the entries as “online” or “offline”*; Detect the target brand*	YES*
OpenPhish	YES*	URL; Time	N/A*	NO*
PhishStats	YES	URL; Date	N/A*	NO*
Piracema	NO*	URL; register_time; page_content; hosting_service; target_brand; shorterner_service; whois_creation_time	Removes the duplicated entries; analyzes the false positives and negatives entries; available the page content (snapshot); analyzes the domain reputation (detects hosting service, domain registration, and domain age); detects the target brand through the NLP.	YES*

Initially, the platforms have a common way of obtaining new records, where PhishTank and PhishStats allow users to submit and view phishing URLs that are updated daily on the websites. In OpenPhish, the submission of new complaints is via e-mail. Piracema does not allow the community to report malicious URL because its registry base essentially comes from other platforms. However, we do intend to implement this functionality in the future.

Another point is the number of features and aspects analyzed in each platform. For example, PhishTank and PhishStats have a “date” field that tells you the date and time when phishing occurred. However, OpenPhish has the “time” field that provides only the time of the occurrence, which leads to the assumption that the day of the occurrence would be the date the URL was published on the platform. As a differential, Piracema offers additional information compared to other databases, such as page_content, whois_creation_time, and other fields exposed in the “Data offered” column of the Table 11.

Regarding the pre-processing carried out in the repositories, PhishTank is the only one that performs some data analysis. For example, it describes which pages are “online” or “offline”, as well as pages that have been confirmed as threatening or benign. The problem is that, due to the volatility of phishing, it is not possible to track this information in real-time. So it’s not uncommon to find pages marked “online” but no longer available.

Another problem, specific to PhishTank, is the inconsistency in cataloging the target of phishing attacks, where the “target” field had a generic value (“Other”) or a tag that was not the true target of the attack. For these cases, our proposal circumvented the situation through NLP techniques, making the field reliable and consistent. More details on the applied NLP technique are available in Silva et al.^18^ study.

Duplicate records were also found, whether caused by the same URL in different repositories or even two or more URLs registered in the same repository. For this reason, during the construction of the Piracema database, care was taken to identify these duplicates and remove them from the data through an analysis based on *hash* collision.

Finally, the Table 11 also exposes the presence or absence of an API to query the platform records. It is possible to observe that the OpenPhish or PhishStats platforms do not have this feature, at least for free and without limitations. In PhishTank, there is only a limitation on the limit of *bandwidth* per *api_key*. The Piracema platform has an API to check records, including to detect whether a given URL is malicious or not, through a classification model based on machine learning. Queries can be carried out on the website itself or through an extension for Google Chrome and Mozilla Firefox. More details about the classification model can be found in Silva et al.^18^ study.

From the phishing behaviors that are described in the work by Silva et al.^18^ and observing the data and observing the data and its structure described in the Tables 9 and 10, it is possible to analyze aspects of phishing such as:

- Trustworthiness: Textual and visual identity of a brand. Examples: logos, template and keywords.
- Obfuscation: Concealing details or subterfuge of information. Examples: behavior simulations via JavaScript.
- Propagation: Multiplicity and cloning. Examples: (content hash collision, variables that modify the URL to bypass, hosting and more exploited tld (among other information that can be extracted from the URL).
- Seasonality: Calendar events. Examples: planned events, celebrations, emergency situations.
- Volatility: Reputation based on Lifecycle. Examples: analysis of activity via the WHOIS protocol (via the URL).

As for the static aspects, such as the URL, hosting service, target brand, among others, such information provides a relevant data set for the detection of targeted phishing, that is, with high trustworthiness. Another point is the sample diversity present in the database, which favors support for studies aimed at proposals for new solutions for phishing prediction based on static behaviors, as in the study by Silva et al.^17^, which did logistic regression to observe behavior patterns in the URL.

Regarding the dynamic aspects, although only 14.38% (135,542) of the records provide the source code of the page, it is still a satisfactory amount for researchers to observe patterns of dynamic behaviors such as homographic attempts, explorations by seasonality and behavior techniques forged. In addition, the data obtained by WHOIS present dynamic features of phishing related to the time it has been in operation, according to a study by Silva et al.^18^ who did logistic regression to look at patterns of phishing lifecycle behavior.

A partir das contribuições mencionadas acima, ainda é possível afirmar que apesar da base Piracema ser uma abordagem diferente e apresentar informações adicionais sobre o conteúdo das páginas maliciosas, a mesma não invalida a existência de outros repositórios, uma vez que o Piracema é constituído a partir de ocorrências das outras 3 fontes de registros phishing mencionadas neste trabalho. Dito isso, a base de dados proposta será atualizada futuramente e irá recorrer às mesmas plataformas apresentadas e, possivelmente outras, e dará continuidade aos mesmos aprimoramentos apresentados e novos que possam surgir. Dessa forma, a proposta se mostra relevante para a literatura por ser uma opção rica em pré-processamento de informações, podendo servir diretamente como auxílio para propostas que visam construir datasets com intuito de mitigar ataques de phishing, como é o caso da pesquisa de Orunsolu et al.^24^, que analisa as features de ocorrências originadas do PhishTank para dar suporte ao seu modelo preditivo, bem como os trabalhos de Tang et al.^28^, He et al.^29^, Qi et al.^30^ e Ma et al.^31^, que utilizam técnicas de machine learning para aprimorar a detecção de ameaças virtuais.

## Data Availability

All information about the phishing records present in the base is available on our website (https://piracema.io/repository). On the website, it is also possible to navigate between records, observing fraud elements such as URL, registration time and other information in a more visual and interactive way. All files with content extracted from malicious pages are available for download from https://bit.ly/piracema-raw and currently, the files are kept in Zip file format. ***The password for zip file: ScientificReports2022@#$***.
